# Supplementary material for: Are carbon emissions trading and green financial instruments synergistic? -Comprehensive quantitative research based on content analysis
Source: PLoS One. 2024 Mar 7;19(3):e0298601. doi: 10.1371/journal.pone.0298601 (PMC10919601; doi:10.1371/journal.pone.0298601)
Supplement: S1 Data — (ZIP) [file pone.0298601.s001.zip › Data/地级市Z_F工作报告.zip!/░▓╗╒/╦▐╓▌╩╨/└┤╫╘╣1⁄2╓┌║┼í╛┬φ┐╦╩2╛▌═°í┐_2005.docx]

2005年2月26日宿州市第二届人民代表大会第二次会议

市 长  唐承沛

各位代表：

现在，我代表市人民政府，向大会作政府工作报告，请予审议，并请市政协委员和其他列席人员提出意见。

一、过去一年的回顾和今年工作的基本要求

2004年，是新一届政府的开局之年，也是我市积极适应宏观调控，抢抓机遇，加快发展的一年。在中共宿州市委的领导下，全市人民开拓进取，扎实工作，全面完成了市二届人大一次会议确定的目标任务。全市生产总值达到279亿元，增长14%。财政收入12.6亿元，按可比口径增长12.7%。城镇居民人均可支配收入7013元，农民人均纯收入2130元，分别增长13.2%和24.8%。各项社会事业也取得了长足进步。一年来，主要抓了六个方面的工作：

一是着力培育发展后劲。正确处理加强宏观调控与加快发展的关系，把宏观调控期作为新一轮大发展的调整期和准备期，把项目建设作为加快发展的长远之策，切实保护好、引导好、发挥好各方面加快发展的积极性。结合本届政府五年发展任务，制定出台了煤电产业、民营经济、诚信宿州建设等10多个专题实施方案，进一步明确了发展方向。研究提出“6＋3”工程，规划储备了230多个重大项目，总投资926亿元，为加快发展构筑了坚实平台。切实采取有效措施，努力缓解宏观调控带来的土地、资金制约，千方百计扩大投入，全社会固定资产投资67.5亿元，增长18.5%。工业项目建设年成效显著，规模以上工业企业完成增加值27.7亿元，增长21.3%。丰原生化环氧乙烷、砀山果蔬速冻罐头、明丽水泥、光明木材大市场等一批项目相继投产。引进了博洋集团、锦丰集团、三狮集团等一批知名企业。汇源#4机组、中南钢铁、申博木业、界沟煤矿建设加快。宿州电厂一期工程、丰原小麦深加工前期工作取得重大进展。明睢和泗永高速公路、宿淮铁路、淮水北调、湖洼地治理、林浆纸一体化等重大项目筹备工作有序推进。

二是持续扩大对外开放。把招商引资作为推动经济发展的突破口，强化目标管理，落实“一岗双责”，精心组织了苏北鲁南学习考察、皖鲁经济合作洽谈等招商活动。对外坚持不懈地抓好浙江、广东等重点区域的小分队招商，对内全面推进与皖北煤电集团、中煤三建公司等驻宿大企业的合作，招商规模实现新的突破。全年实际到位内资30.5亿元，利用外资2330万美元。民间投资日趋活跃，新增私营企业760家，个体工商户9001户，注册资本7亿元。外贸出口完成2159万美元，同比增长6.3%。深入开展“双项活动”和“四增一树”学教活动，集中实施了重点企业、重点项目、重点工程周边环境、城区违法建设等专项整治，严肃查处了一批损害经济发展环境的案件，社会各界对优化经济发展环境的信心普遍增强。

三是加速推进城市建设。把加快城市化进程作为统筹城乡发展、融合工业文明、活跃经济全局的重大举措，着力提升城市的功能和形象。城区规划不断加强，政务中心等3项控制性规划、沱河大市场等32项修建性详规编制完成。城区路网建设全面推进，汴河中路和汴河路立交桥改造工程顺利完工，淮河路、三八路、银河二路、纺织东路建成通车，外环路、宿怀路、张仙庙路中段、银河一路、汴河西路及雪枫广场改造正在紧张施工。宿州经济技术开发区基础设施建设加快，农业科技示范园和宿州循环经济示范园规划全面启动。城市综合开发势头良好，当年竣工房地产面积58万平方米。行政中心主体封顶，光彩城大市场、城隍庙商城等项目建设加快。实行市区联动，深入开展脏乱差、占道经营、城区交通秩序等专项整治，城市文明创建水平有新的提升。

四是全面发展农村经济。认真贯彻落实中央1号、省委8号文件精神，在政策、气候等多种利好因素作用下，粮食大幅增产，农民大幅增收。全年粮食总产332万吨，油料总产35万吨，创1997年以来最好水平。畜牧业克服禽流感影响平稳发展，肉类总产44万吨，增长7.2%。农业产业化取得积极进展，省市级产业化龙头企业发展到36家，农村经济合作组织和专业协会发展到324家。全年劳务输出总量超过85万人，比年初净增15万人。农田水利基本建设成效显著，奎濉河五期治理顺利完成，水毁工程全部修复。绿色长廊工程建设和农机化综合水平继续保持全省领先地位。全面落实“两减免三补贴”政策，农民人均政策性增收60元。

五是统筹经济社会协调发展。就业、再就业工作力度加大，全年新增就业岗位2.3万个，城镇登记失业率控制在4.2%。积极推进“三条保障线”衔接，低保基本实现了应保尽保、分类施保。农村科技富民工程、工业技术创新工程深入实施。教育事业健康发展，宿州学院成功挂牌，宿州高专正在筹建。文化事业繁荣活跃，文艺创作在省内外一系列重大赛事中获奖，成功举办了首届中国灵璧石文化节。竞技体育、群众性体育成果丰硕，我市运动员在第十二届雅典残奥会上获得4枚金牌。红十字会工作受到国家表彰，疾病预防控制体系和医疗救治体系建设进展顺利。计划生育专项清理成效明显，基础工作进一步夯实。生态市建设开始启动，砀山县被列为省首批综合示范基地。生产生活资料市场秩序有效规范，安全生产形势相对平稳。精心构筑社会治安防控体系，妥善处置涉法上访，社会形势总体稳定。精神文明、基层民主建设得到加强，萧县龙城镇姬村被命名为全国民主法治示范村。双拥、人防和民兵预备役建设卓有成效。妇女儿童、老年人和残疾人的权益得到较好保障。经济普查和第二轮方志修编工作顺利启动。人事编制、广播电视、新闻出版、民族宗教、外事、物价、对台、气象、地震等工作也取得新的进展。

六是切实加强政府自身建设。围绕建设学习型、服务型、效率型、责任型政府，修订完善了《宿州市人民政府工作规则》，进一步规范了政府工作程序。坚持定期向市人大常委会报告工作，向市政协常委会通报政情，密切与各民主党派、工商联、无党派人士和人民团体的联系。全年办复人大代表议案、建议138件，政协委员提案229件，办结率均达100%。积极贯彻落实《行政许可法》，改革行政审批，扩大政务公开，改进政风评议，有效促进了行政提速。积极履行政府公共管理职能，启动突发性公共事件应急预案的制定工作，应对突发事件的能力明显提高。严格落实政府廉政建设责任制，强化领导干部经济责任审计，狠刹部门和行业不正之风，各级机关作风有了新的转变。

各位代表，在中央实施宏观调控、外部经济环境发生重大变化的情况下，我们仍然取得了较快的发展。这主要得益于市委的正确领导，得益于全市广大干部群众的奋力拼搏，得益于各级人大、政协对政府工作的鼎力支持。在此，我谨代表市人民政府，向全市广大人民群众，向驻宿人民解放军、武警官兵和省属企事业单位干部职工，向各民主党派、工商联、人民团体和离退休老同志，向所有支持、参与宿州建设的海内外客商表示衷心的感谢，致以崇高的敬意！

各位代表，2004年，我们虽然在推进宿州崛起的道路上迈出了重要一步，但同时也面临不少困难和问题。主要表现在：一是发展的基础尚不稳固。经济增长的恢复性成分较多，主要经济指标与全省平均水平差距较大；煤电油运供需矛盾以及土地、资金约束尚未缓解，影响经济增长的不确定因素增多。二是发展中的热点难点问题较多。县域经济活力不强，县乡财政普遍困难；农民增收基础不稳，社会保障和就业任务较重；社会矛盾的化解机制不够健全，维护稳定的压力依然较大。三是政府工作还有不少薄弱环节需要加强。政府职能转变滞后，依法行政水平亟需提高，特别是少数干部思想不解放、精力不集中、工作不落实、服务不到位的现象，以及形式主义、官僚主义作风还不同程度存在。总体上看，宿州发展仍处在艰难的爬坡阶段，处在打基础的关键时期。我们必须自加压力，负重奋进，团结拼搏，合力攻坚，不辜负全市人民的期望。

各位代表，今年是全面实现“十五”目标，衔接“十一五”发展的关键一年。做好各项工作，对于宿州加快发展、奋力崛起，全面建设小康社会，意义十分重大。根据中央、全省经济工作会议和市委二届三次全会精神，今年政府工作的基本要求是：认真落实科学发展观，积极适应宏观调控政策，坚定不移地以农业为基础，以工业化为核心，以大开放为主战略，以招商引资为突破口，坚持开放兴市、工业强市，全面实施“6＋3”工程，着力提高工业化、城镇化、农业产业化和城乡居民生活水平，促进经济社会全面协调可持续发展。

主要预期目标是：生产总值增长8.5%左右，财政收入按可比口径增长5.9%，全社会固定资产投资增长25%，城镇居民人均可支配收入增长8%，农民人均纯收入增长5.6%，城镇登记失业率控制在4.5%以内，人口出生率控制在13‰以内。

实现上述目标，从我市现有生产力水平和发展基础来看，尚需付出艰苦努力，在实际工作中还要力争发展得更快更好，有条件的县区应当有更高的目标。对此，我们必须保持奋发有为的精神状态，积极应对各种新的考验，创造性地开展工作，确保完成各项预定目标。

二、抢抓机遇，乘势而上，奋力推进宿州崛起

推进宿州崛起，改变落后面貌，关键在于抓投入，上项目，增后劲，提升经济总量。当前，宏观经济走势继续向好，沿海地区生产要素加快转移，国家对中部地区扶持力度加大，我市资源优势、区位优势进一步凸显。全市上下团结和谐，广大干群人心思上，发展的能量正在加速释放。在2005年工作当中，我们必须进一步抓住难得的发展机遇，倍加珍惜来之不易的发展势头，切实增强奋力崛起的紧迫感和危机感，全面动员，全力以赴，千方百计保持经济快速增长。

全面实施“6＋3”工程，即推进煤电产业、农产品生产加工、加工制造、劳务供应、商贸流通、文化旅游六项产业基地和道路交通、农田水利、社会公共事业三项基础设施建设，是市委、市政府在深入分析国家产业政策，准确把握市情的基础上，作出的一项重大战略决策。“6＋3”工程以项目建设为核心，覆盖经济建设和社会发展各个方面，是推进经济结构战略性调整的重要抓手，实现经济社会协调发展的重要载体，对于锻造我市工业化脊梁，加快城镇化和农业产业化进程，提升我市综合竞争力具有至关重要的意义。今后一个时期，要把实施“6＋3”工程作为振兴宿州经济、振奋民心士气的关键性工程，建立调度考核管理机制，着力形成上下联动、合力推进的工作氛围。

坚持不懈地推进项目建设。全面加快在建项目进度，积极创造良好环境，确保汇源#4机组、界沟煤矿等在建项目年内建成投产。快节奏做好各项准备工作，争取宿州电厂、丰原小麦深加工、龙王庙煤矿等项目开工建设。认真抓好项目前期准备，千方百计落实投资主体，落实审批手续，落实土地供应，推动明睢和泗永高速、淮水北调、宿淮铁路、林浆纸一体化等重大项目前期工作取得突破。加强项目规划储备工作，围绕“6＋3”工程，发动全社会力量参与项目的研发与策划。特别要依托皖北煤电集团、中煤三建公司等驻宿大企业的资金、技术、人才和市场优势，研发提报重大项目。适应国家产业政策，瞄准国债资金投向，积极争取农田水利、科教文卫、道路交通、环保治污、循环经济等政策性项目。做好新一轮土地利用总体规划修编，确保新增建设项目用地。对所有入库项目，委托专业机构编制深度可研报告，扎实做好包装、论证等基础工作，切实增强项目的可行性和操作性。强化长远发展战略的研究和谋划，以项目建设为支撑，制订“十一五”发展计划。

全力以赴抓好招商引资。坚持项目建设和招商引资相结合，通过招商引资落实投资主体，筹集项目资金。继续强化目标管理，严格落实“一岗双责”，确保全年引进内资40亿元，外资2650万美元。加强招商队伍建设，集中力量抓好小分队定点、定向招商。强化招商人员业务培训，不断提高招商能力。现有市政府驻外办事处一律调整为招商联络处，在沿海发达地区增设若干招商联络处，进一步扩大驻点招商范围。积极探索网上招商方式，培育招商中介组织，实现招商引资市场化和专业化。做好外来企业的服务工作，推进以商招商、以外引外。调整完善招商引资优惠政策，主攻长三角、珠三角、山东半岛等重点区域，加快实现项目和资本的对接。在提升工业领域招商规模、引资质量的同时，积极推进农业、旅游和城市基础设施的招商。打造各类招商载体，集全市之力支持宿州经济技术开发区发展，凡涉及开发区的事务，市直部门实行“委托办理”、“见章盖章”或“柜台前移”方式，支持开发区创新管理体制，完善服务功能，增强吸纳和辐射能力。推动农业科技示范园向农业开发区发展，积极申报宿州循环经济示范园，尽快形成“一区两园”相互呼应、联动发展的格局。

加快提升城市发展水平。坚持建设与管理并重，完善城市功能，塑造崭新形象，不断增强城市对经济发展的承载能力。切实抓好宿城基础设施建设，提高宿城在全市经济发展中的地位。以高速公路出入口为顶点，以三八路、高开路为依托，以经济技术开发区、政务中心为两翼，加快建设“L”型城市新区，促进人口从老城区疏散，建设向新城区集中，工业向开发区集中。深化实施城市总体规划，编制完成高开路区域等控制性详规、沱河景观带和合徐高速公路出入口环岛规划，以及城市交通、绿化等专项规划，确保城市建设有规可循。继续加快城区主干道路建设，打通关键节点，全面完成外环路、宿怀路、银河一路、浍水西路、汴河西路建设，建成金海北路、胜利西路、濉河路、西昌北路和南外环路连接线，启动高开路、淮海南路、西昌南路、张仙庙路北段建设，力争经过两年左右的努力，基本建成宿城主干路网。启动沱河穿城段治理和市府广场建设，完成运粮河治理和雪枫广场改造，稳步扩大城区绿地面积。开工建设宿州垃圾处理场，新建改建一批垃圾中转站和高标准公厕。实施老城区主干道精细化管理，完成胜利路文明示范街改造。实行市区共建，启动小街小巷五年治理计划。规范地名管理，完善城市路标设置。强化城区交通秩序专项整治，加大脏乱差治理力度。高标准修编县城和乡镇总体规划，充分发挥中心镇示范作用，重点抓好县城建设，提升城镇对县域经济的辐射带动能力。

坚定不移地抓好农村经济结构调整。高度重视发展农村经济，坚持以农民增收为核心，以市场需求为导向，围绕与大工业的联姻和龙头企业的配套，调整优化种植结构，稳定粮食综合生产能力。突出发展畜牧养殖、特色农业和林业经济，形成优质高效农业产业带。健全农产品质量安全检验检测体系，实施农业标准化生产，提升农产品竞争力。加快推进农业产业化经营，重点扶持一批带动力较强的龙头企业。积极推广萧县发展农村专业合作经济组织的试点经验，进一步壮大农村经纪人和致富带头人队伍，增强农民进入市场能力。强化农民职业技术培训，规范劳动力市场，畅通信息网络，健全输出体系，打造宿州劳务品牌，力争全年外出务工突破100万人。抓住国家大力支持农业的机遇，积极争取国家和省支农资金，认真落实“三补一免”等各项政策。全面贯彻土地承包法，促进土地经营权有序流转。抓好县乡公路通达工程、农田水利、农村沼气、农村人畜安全饮水、数字化气象雷达等农业基础设施建设，改善农业生产条件，提高农业抗御自然风险的能力。广大农村基层干部工作辛苦，任务繁重，各级政府要更多地给予体谅、关心和支持，更好地保护并调动他们做好农村工作、发展农村经济的积极性。

深化以市场为取向的各项改革。加大国有企业改革力度，鼓励现有优势企业通过产权置换、嫁接重组等途径，靠大联大，做优做强。对现有困难企业，稳步实施依法破产，整合盘活存量资产。放手发展民营经济，出台扶持民营经济发展的优惠政策，引导民间资本进入法律法规未禁止的行业和领域，进一步营造全社会鼓励、支持民营经济发展的氛围。深化城市基础设施维护和投资体制改革，推进城市污水处理、市政设施维护、园林绿化、道路保洁市场化，鼓励社会力量投资、垫资兴建道路和其他公共设施，公开拍卖城市道路、桥梁命名权和市政设施广告发布权，实现城市资源效益最大化。健全公共财政框架，强化综合预算、国库集中支付等改革措施，提高财政资金的使用效益。稳妥推进市县区政府机构改革和乡镇区划调整，巩固乡镇事业单位人员分流成果。继续深化科技、教育、文化、卫生等方面的改革，增强社会发展活力。

努力提高经济运行的质量和效益。加强煤电油运的综合协调，确保重点企业、重点产品的生产要素供给。完善中小企业发展的资金、技术和政策服务体系，支持企业加快技术改造，增强自主研发能力，壮大中小企业集群。加强财源建设，加大骨干企业、利税大户的培植力度，增强重点财源对财政的支撑能力。优化出口商品结构，积极落实各项外贸促进政策，努力扩张出口总量。创建良好金融环境，建立信贷投向和产业发展的双向传导机制，增加有效信贷投入。抓好全市商业网点规划修编，依托华夏、美惠多等骨干流通企业，引进知名商业企业，提高城市商业档次。积极培育电子商务、物流配送、连锁经营等新型业态，壮大社区服务、中介咨询、物业管理等新兴服务业。加快县域经济发展，支持各县区发挥比较优势，加强资源开发，突出发展煤炭开采、农副产品加工以及劳动密集型产业和配套产业，逐步形成各具优势和特色的支柱产业。

三、以人为本，统筹发展，努力建设和谐宿州

推进宿州崛起，要以社会和谐为保障，让人民群众从经济发展中得到更多的实惠，在社会进步中享受更多的文明成果。各级政府及其工作人员要牢记执政为民的宗旨，切实维护群众利益，注重发展各项社会事业，努力创造一个充满活力、协调发展、稳定有序的社会局面。

加大就业和社会保障力度。把促进就业作为经济社会协调发展的重要目标，认真落实再就业各项扶持政策，支持下岗失业人员自谋职业、自主创业和灵活就业。加强城镇新增就业人群的培训，推进就业援助制度化，确保全年新增就业岗位2.1万个。进一步健全社会保障体系，依法推进社会保险扩面征缴和社会保险关系接续，确保养老金、失业金按时足额发放，提高企业退休人员社会化管理水平。完善城市低保工作，切实做到动态管理下的应保尽保。

妥善解决好关系群众切身利益的问题。扎实做好矿区、城镇失地农民安置，依法实施对城镇居民的拆迁补偿，认真落实改制企业职工的经济补偿政策，建立防范拖欠农民工工资的工作机制。加强农村特困救助和医疗救助，做好五保供养。促进残疾人事业发展，高度关注老年人和妇女儿童的权益保护。努力解决特困家庭、特困人群的生产生活困难，保证弱势群体的烦事、难事、苦事有处倾诉，有人解决。整治公路“三乱”，规范医药购销、教育收费等行为，坚决纠正部门和行业不正之风。加强工矿企业、交通、消防等安全基础工作，防范重特大事故的发生。进一步规范生产生活资料市场秩序，确保人民群众食品、药品安全。

加快发展各项社会事业。高度重视人力资源开发，加强企业家队伍和科技人才队伍培养，注重引进各行各业领军人物和拔尖人才，努力打造能够充分发挥各类人才作用、体现人才价值的事业平台，激发人才创业潜力。加快构建现代教育体系，完善农村义务教育管理体制，大力发展职业教育，规范发展民办教育。积极申办技师学院，抓好宿州高专筹建和卫校新校区建设，支持宿州学院、宿州职业技术学院提高办学质量，扩大办学规模。加快公共卫生体系建设步伐，启动市立医院门诊楼建设，基本建成市疾病预防控制体系和医疗救治体系。加快市体育馆和体育中心建设，发展文化和体育产业，丰富城乡群众文化生活。强化重点污染源治理，抓好生态市建设。努力稳定低生育水平，着力解决出生人口性别比偏高问题。继续做好经济普查、外事侨务、新闻出版、对台、地震、气象、档案、地方志等工作。

加强民主法制建设。抓好普法教育，完善法律援助体系，增强全民法制观念。积极稳妥推进司法体制改革，加强社会治安综合治理，严厉打击各类刑事犯罪和经济犯罪，努力创建“平安宿州”。加强《信访条例》宣传，依法规范群众上访行为，及时排查调处社会矛盾纠纷。加强城镇社区基础建设，组织好第六届村民委员会换届选举，增强基层政权管理能力。积极支持工会、共青团、妇联等群众团体依照法律和各自章程开展工作。全面贯彻党的民族宗教政策，依法管理民族宗教事务。进一步做好国防动员、民兵预备役、人防和优抚安置工作，争创全国“双拥模范城”五连冠。

塑造良好宿州形象。加强诚信宿州建设，着力构建以政府诚信为先导、企业诚信为重点、个人诚信为基础的信用体系。各级政府及其部门要慎言重诺，取信于民，带头营造“守信为荣、失信可耻”的社会环境。以行业、社区为重点，深入开展群众性精神文明创建活动，加强市民文明行为的教育，不断提高全市人民的科学文化素质、思想道德素质和城市意识。弘扬敢试、敢闯、敢冒的创新精神，营造鼓励创业、包容失误的社会氛围，激发全社会的发展活力。大力倡导“建设宿州、爱我宿州”的良好风尚，引导广大干部群众从自身做起，从小事做起，多做有利于宿州的事，多为宿州增光添彩。切实改进和加强对外宣传工作，充分发挥政府网站、广播电视、报刊等舆论阵地作用，加强与主流媒体的联系与沟通，及时务实地回应新闻媒体的批评和曝光，充分展示宿州人民诚实守信、文明开放、干事创业、奋力崛起的良好形象。

四、转变职能，优化服务，切实增强政府行政能力

推进宿州崛起，是宿州人民的最大愿望，也是全市人民赋予我们的光荣职责。各级政府及其工作人员要顺应民心民意，把握发展大局，认真贯彻落实党的十六届四中全会精神，以增强行政能力为核心，大力推进科学行政、民主行政、依法行政，不断提高驾驭市场经济的能力和管理社会事务的水平。

深入推进依法行政。各级政府及其部门要牢固树立依法行政观念，认真实施《行政许可法》和国务院依法行政纲要，严格按照法定权限和程序行使权力，善于运用法律手段管理经济和社会事务，确保政府一切工作都在法律框架下运行。深化行政审批制度改革，认真清理行政许可事项、许可主体，切实规范行政许可收费及管理。严格规范性文件的制定程序，建立市政府法律顾问组。完善各级行政服务中心功能，减少审批环节，提高审批效率。加强行政执法监督，健全行政执法主体和行政执法人员资格制度，严格执法过错责任追究，确保公正执法、文明执法。各级政府要主动接受人大及其常委会依法监督和人民政协民主监督，自觉接受广大群众和新闻媒体的监督，认真办理人大代表议案、建议和政协委员提案。实行政务公开，提高政府工作透明度。

强化决策实施能力。在宏观调控中加快经济发展，是对各级政府行政能力的严峻考验。我们不仅要科学谋划宿州的长远发展大计，更要着力抓好已定部署的贯彻实施。要大兴求真务实之风，精简会议、文件，改进会风、文风，开短会、讲短话，减少事务性活动，杜绝形式主义的检查评比。切实加强效能监察和政务督查，严禁推诿扯皮、阳奉阴违，确保各项工作有部署、有检查、有反馈。坚持从实际出发，做好结合的文章，把宿州发展的思路和要求传达到广大人民群众之中，充分调动各方面干事创业的积极性和创造性。各级政府及其部门在加快发展的过程中，不但要善于提出思路和措施，还要勇于挑担子，敢于担责任，形成一级支持一级，一级保护一级，一级带动一级抓发展、促崛起的工作局面。

持续优化发展环境。营造良好的发展环境，是政府行政能力的具体体现。各级政府及其部门要牢固树立环境就是竞争力的意识，把履行职能与维护大局结合起来，把管理与服务结合起来，把政策的原则性与操作的灵活性结合起来，努力为企业营造公平竞争的市场环境，为投资者营造宽松舒心的发展环境。进一步加强对各级公务员特别是行政执法人员的教育，切实解决粗暴执法、多头执法问题，克服行政机关中态度冷硬、作风散漫、吃拿卡要等不良现象。出台关于损害经济发展环境的处理办法，加大投诉案件的查处和责任追究力度。改进政风、行风评议，定期组织企业对政府部门的服务质量和服务效率进行评议。加强对社会环境的管理，严厉打击非法阻工、强装强卸、违章建设行为，为重点工程的建设创造良好外部环境。

切实加强政风建设。面对全市人民加快发展的强烈渴望，面对区域经济竞相发展的逼人态势，我们的各项工作，干得再多也不算多，抓得再好都有差距。各级政府及其工作人员要切实增强事业心和责任感，始终保持昂扬奋进、排难而上的精神状态，坚决克服消极畏难、无所作为的不良倾向。坚持求真务实，力戒官僚作风，力戒形式主义，多做打基础、管长远的工作，多办得民心、顺民意的事情。牢固树立正确的权力观，坚决反对讲排场、比阔气、铺张浪费等恶习。按照标本兼治、综合治理、惩防并举、注重预防的要求，健全重大事项报告、任期经济责任审计等廉政制度，落实廉政建设责任制。在各级政府机关深入开展保持共产党员先进性教育活动，教育广大公务员树立正确的理想信念和高尚的道德情操，爱岗敬业，乐于奉献，始终保持公仆本色，赢得人民群众对改革发展的更大支持，为宿州崛起提供更加坚实的保障。

各位代表，宿州正处于加快发展、奋力崛起的关键时期。唯有振奋精神，迎难而上，才能抓住机遇，实现崛起；唯有求真务实，干事创业，才能不辱使命，不负重托。让我们在中共宿州市委的领导下，紧密团结在以胡锦涛同志为总书记的党中央周围，开拓创新，锐意进取，为全面完成今年各项任务，加快实现“两个跃升、一个崛起”的宏伟目标而努力奋斗！
